# The associations of serum S100A9 with the severity and prognosis in patients with community-acquired pneumonia: a prospective cohort study

**DOI:** 10.1186/s12879-021-06020-y

**Published:** 2021-04-07

**Authors:** Hong-Yan Liu, Hui-Xian Xiang, Ying Xiang, Zheng Xu, Chun-Mei Feng, Jun Fei, Lin Fu, Hui Zhao

**Affiliations:** 1 Respiratory and Critical Care Medicine, Second Affiliated Hospital of Anhui Medical University, Furong Road no 678, Hefei, 230601 China; 2 Department of Toxicology, Anhui Medical University, Hefei, 230032 China

**Keywords:** Community-acquired pneumonia, S100A9, Inflammatory cytokines, CAP severity score, Biomarker

## Abstract

**Background:**

Previous studies found that S100A9 may involve in the pathophysiology of community-acquired pneumonia (CAP). However, the role of S100A9 was unclear in the CAP. The goal was to explore the correlations of serum S100A9 with the severity and prognosis of CAP patients based on a prospective cohort study.

**Methods:**

A total of 220 CAP patients and 110 control subjects were recruited. Demographic and clinical data were collected. Serum S100A9 and inflammatory cytokines were measured.

**Results:**

Serum S100A9 was elevated in CAP patients on admission. Serum S100A9 was gradually elevated parallelly with CAP severity scores. Additionally, inflammatory cytokines were increased and blood routine parameters were changed in CAP patients compared with control subjects. Correlation analysis found that serum S100A9 was positively associated with CAP severity scores, blood routine parameters (WBC, NLR and MON) and inflammatory cytokines. Further, logistic regression analysis demonstrated that there were positive associations between serum S100A9 and CAP severity scores. Besides, the prognosis of CAP was tracked. Serum higher S100A9 on the early stage elevated the death of risk and hospital stay among CAP patients.

**Conclusion:**

Serum S100A9 is positively correlated with the severity of CAP. On admission, serum higher S100A9 elevates the risk of death and hospital stay in CAP patients, suggesting that S100A9 may exert a certain role in the pathophysiology of CAP and regard as a serum diagnostic and managing biomarker for CAP.

## Introduction

Pneumonia is a primary cause of morbidity and mortality all over the world, especially in children and in adults more than 60 years. Every year, 450 million people suffered from pneumonia and 1.3 million cases died of pneumonia [1–3]. *Streptococcus pneumoniae* (pneumococcus) is one of the commonest bacterial pathogens-inducing community-acquired pneumonia (CAP), which results in substantial health and economic burden [4, 5]. Previous studies found that CAP accounts for more than 60,000 deaths, 1.2 million hospitalizations, 2.3 million emergency department visits and $10 billion in hospital costs in United States yearly [6, 7]. In order to reduce the mortality and prevent CAP clinical deterioration, it is very necessary to diagnose the disease and evaluate the severity of CAP at the time of disease onset. Nevertheless, early recognition and evaluation the risk of CAP are difficult. The sensitivity and specificity of different biochemical markers and laboratory testing are variable and largely limited [8]. Therefore, additional new biomarkers are essentially needed to evaluate the severity and simplify the diagnosis progress.

S100A9, a small Ca^2+^ binding protein recognized as an alarmin, is released by stressed cells: an endogenous danger signal, which promotes and exacerbates the inflammatory response [9]. S100A9 forms a complex with S100A8 (S100A8/S100A9 heterodimer) to exhibit different inflammatory effects via toll-like receptor 4 (TLR4) [10, 11], scavenger receptor CD36 [12] and receptor of advanced glycated end products (RAGE) [13]. Released of S100A9 activates several signaling pathways and exerts important functions in a great deal of cellular processes [10, 14–16]. The previous studies found that S1009A9 is elevated in several diseases, such as neutrophilic inflammation in asthma, insulin deficiency, atopic dermatitis and Parkinson’s disease [17–20]. In addition, our previous study found that S100A8/S100A9 is increased in COPD patients and positively associated with inflammatory cytokines [21]. Therefore, these results indicate that S100A9 may be used as a biomarker for the diagnosis of disease.

An early study revealed that S100A9 was increased in the lung of patients with non-small cell carcinoma [22]. Perinatal inflammation exposure modified lung morphogenesis, elevated the level of S100A9 in fetal mice [23]. TLR4/RAGE signaling was activated and S100A9 was increased under endotoxemia-induced pulmonary inflammation [24]. These data indicated that S100A9 may play an important role in the pulmonary diseases. However, the role of S100A9 protein remains unknown in CAP patients. Therefore, the main purpose of current research was to analyze the correlations of serum S100A9 with the severity and prognosis in CAP patients based on a prospective cohort study.

## Methods

### Subjects

**Fig. 1:**
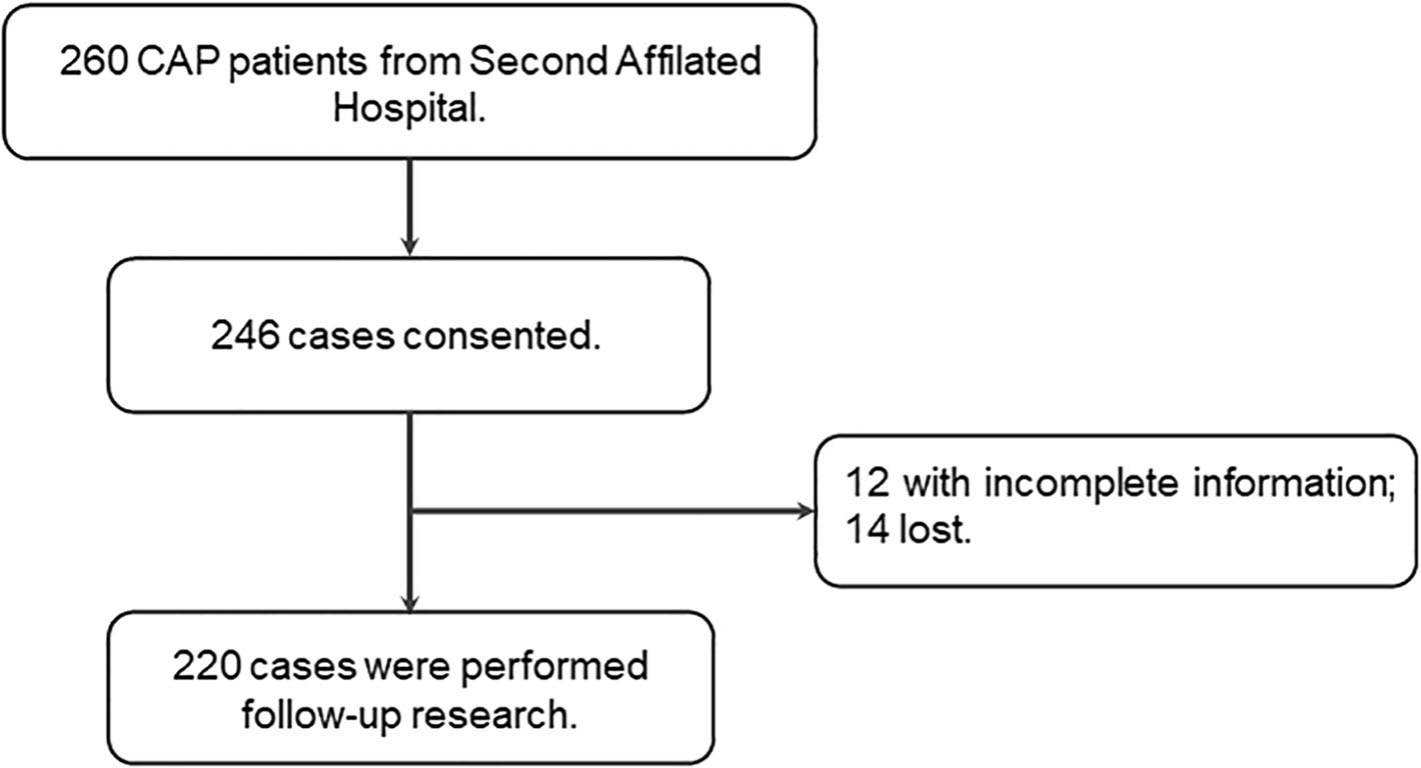
Flow diagram of recruitment and follow-up research in this cohort study

This prospective study was conducted in the Second Affiliated Hospital of Anhui Medical University between May 1, 2018 to June 30, 2020. Altogether, 220 patients with CAP were recruited in the Department of Respiratory and Critical Care Medicine. Demographic and clinical data were collected from Electronic Medical Record System on admission. For CAP patients, the inclusion criteria met the following criteria of CAP [25]; more than 18 years old; signed written inform consents. The exclusion criteria included: severely immunocompromised; pregnant women; patients with other pulmonary diseases, such as serious complications, COPD, lung cancer and asthma; hospital stay was less than 5 days. Healthily control subjects were enrolled from the physical examination center. In the beginning, 260 patients with CAP were enrolled. Two hundred and forty-six cased agreed to perform follow-up research. Twelve patients with incomplete information and 14 patients who were lost were excluded. Finally, 220 patients with CAP involved in this research (Fig. 1). The severity of pneumonia was assessed using CAP severity scores, including pneumonia severity index (PSI), CURB-65 score, CRB-65 score, CURXO score and SMART-COP score. This study was approved by the Ethics Committee in Second Affiliated Hospital of Anhui Medical University. All subjects gave advanced written and oral agreement of their inclusion in this study.

### Enzyme-linked immunosorbent assay (ELISA)

Whole blood was collected at six o’clock in the morning and centrifuged on basis of our previous study [26, 27]. Serum was collected and inflammatory cytokines were measured using enzyme-linked immunosorbent assay (ELISA). Human inflammatory cytokines ELISA kits were obtained from Cusabio, Wuhan, China (https://www.cusabio.com/). Human S100A9 ELISA kits were purchased from Wuhan Colorful Gene Biological Technology Co. Serum S100A9 and inflammatory cytokines were measured according to standard methods [28, 29].

### Statistical analysis

All analysis was conducted with GraphPad Prism 5.0 and SPSS 19.0 software. Clinical features, cytokines and CAP severity scores were compared using Student’s *t* tests, Chi-square tests or Manne-Whitney U tests among different groups. Continuous variables were exhibited median and interquartile range (IQR). Association analysis were performed using linear regression and logistic regression. In order to control confounding factors, age and sex were adjusted. Finally, the multivariate logistic regression analysis was carried out. A two-sided *P*-value of less that 0.05 was regarded as statistically significant.

## Results

### Demographic and clinical data

In total, 220 CAP patients and 110 healthily control subjects were recruited and analyzed in the present study. As shown in Table 1, there was no notable different of age, gender and BMI between CAP patients and control cases. Routine blood test was detected between two groups. We found that white blood cell (WBC) and neutrophil were increased, lymphocyte was decreased in CAP patients. The ratios of platelet-lymphocyte (PLR), monocyte-lymphocyte (MON) and neutrophil-lymphocyte (NLR) were increased in patients with CAP (Table 1). Moreover, liver function and renal function were detected among all cases. As shown in Table 1, except for uric acid, no difference of liver function and renal function was observed in two groups. In addition, inflammatory cytokines were measured. The levels of TNF-α, IL-1β, IL-6 and CRP were elevated in CAP patients. Meanwhile, the severity of pneumonia was assessed with CAP severity scores. Among 220 patients with CAP, the median of PSI, CURB-65, CRB-65 and SMART-COP score was 2.0, 1.0, 94.0 and 2.0, respectively. Additionally, there was 92 (41.8%) severe patients in CAP group (CURXO score) (Table 1).

**Table 1:** Demographic and biochemical characteristics between CAP patients and No-CAP patients

Variables	CAP (*n* = 220)	No-CAP (*n* = 110)	*P*
Age (years)	67.0 (55.0, 80.0)	64.0 (52.0, 75.0)	0.452
Male, n (%)	124 (56.5)	61 (55.0)	0.121
BMI	23.0 (20.2, 25.1)	22.3 (19.8, 24.9)	0.098
WBC (10^9^/L)	6.80 (5.04, 9.35)	5.66 (4.71, 6.81)	<0.05
Neutrophil (10^9^/L)	5.01 (3.13, 7.51)	3.10 (2.41, 3.95)	<0.05
Lymphocyte (10^9^/L)	1.25 (0.86, 1.91)	2.15 (1.86, 2.51)	<0.05
Eosinophil (10^9^/L)	0.07 (0.02, 0.17)	0.11 (0.04, 0.16)	0.061
Monocytes (10^9^/L)	0.45 (0.32, 0.61)	0.35 (0.31, 0.43)	0.060
Basophil (10^9^/L)	0.02 (0.01, 0.03)	0.02 (0.01, 0.03)	0.078
NLR	3.88 (2.02, 8.93)	1.50 (1.31, 2.35)	<0.01
MON	0.32 (0.20, 0.57)	0.19 (0.14, 0.23)	<0.05
PLR	181.4 (117.2, 338.4)	109.2 (89.6, 136.8)	<0.05
ALT (U/L)	19.0 (12.0, 38.3)	17.9 (11.2, 23.3)	0.121
AST (U/L)	24.0 (18.0, 36.0)	23.1 (15.1, 25.1)	0.082
Total bilirubin (umol/L)	10.3 (8.2, 15.1)	14.6 (12.6, 19.3)	0.065
Direct bilirubin (μmol/L)	2.6 (1.8, 3.6)	2.5 (2.2, 3.4)	0.187
Total protein (g/L)	64.6 (59.2, 67.9)	75.6 (66.2, 81.3)	0.241
Albumin (g/L)	33.0 (28.2, 38.2)	44.5 (40.2, 49.6)	0.111
Globulin (g/L)	29.9 (25.5, 35.6)	26.8 (20.1, 34.5)	0.101
Urea nitrogen (mmol/L)	5.21 (4.03, 7.11)	4.56 (3.61, 5.15)	0.387
Creatinine (μmol/L)	58.0 (47.0, 75.5)	60.3 (46.5, 78.0)	0.412
Uric acid (μmol/L)	276.0 (199.0, 333.0)	395.3 (260.5, 462.3)	<0.05
TNF-α (pg/mL)	560.9 (291.8, 1121.8)	62.3 (38.6, 100.3)	<0.01
IL-6 (pg/mL)	70.6 (43.4, 94.3)	28.9 (19.8, 59.8)	<0.01
IL-1β (pg/mL)	361.6 (197.2, 581.2)	60.2 (20.3, 85.5)	<0.01
CRP (mg/L)	43.3 (4.7, 98.2)	8.9 (2.2, 30.1)	<0.01
CURB-65	2.0 (0, 3.0)	N.A	N.A
CRB-65	1.0 (0, 2.0)	N.A	N.A
PSI	94.0 (59.0, 130.0)	N.A	N.A
CURXO [Severe, n (%)]	92 (41.8)	N.A	N.A
SMART-COP	2.0 (0, 5.0)	N.A	N.A

### The levels of serum S100A9 in control subjects and CAP patients

**Fig. 2:**
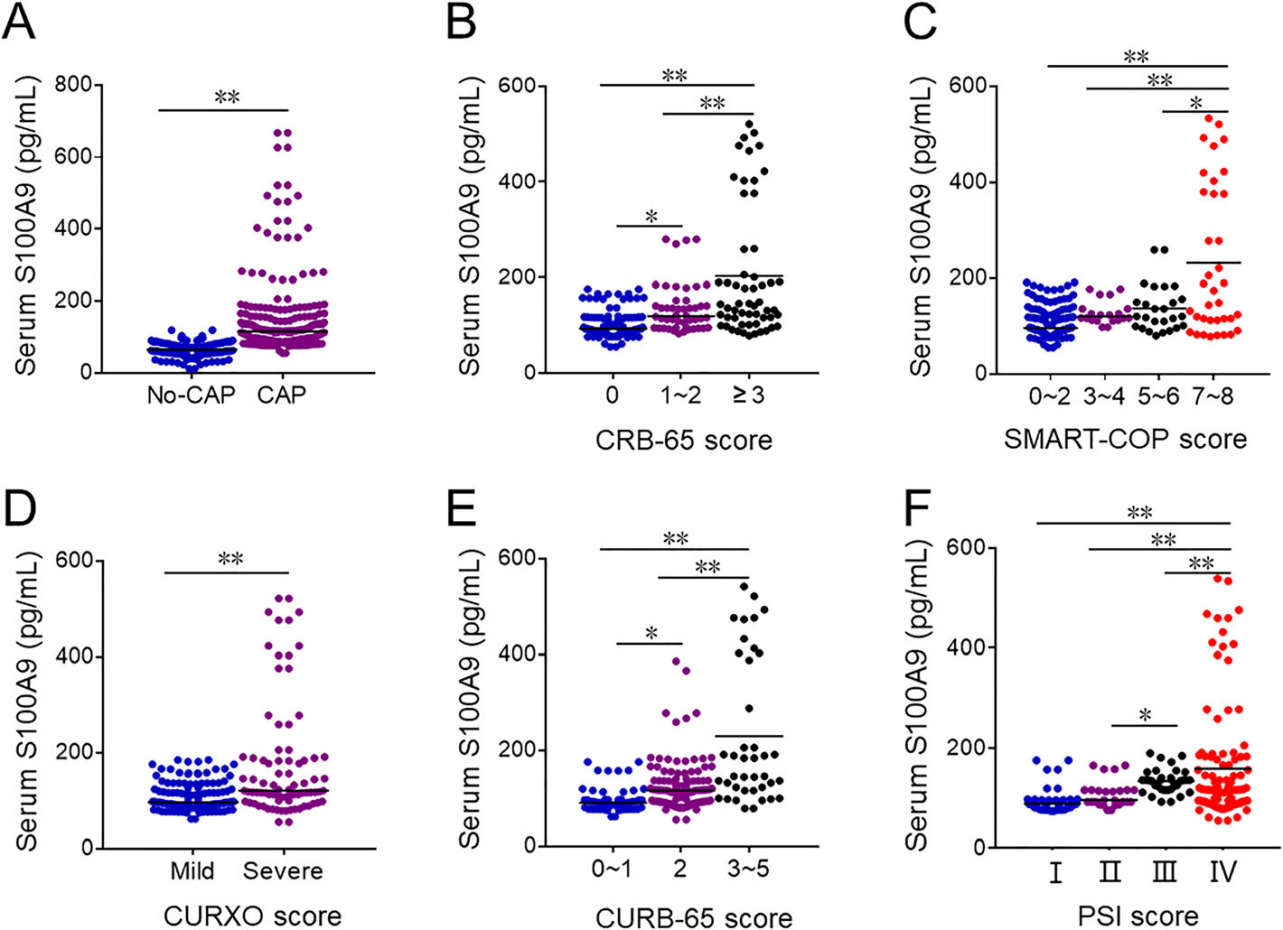
The levels of serum 8-iso-PGF2α in control subjects and CAP patients. **a**-**f** Serum S100A9 was detected with ELISA. **a** The level of serum S100A9 in CAP patients and No-CAP cases. **b** The level of serum S100A9 in different grades of CRB-65 score in CAP patients. **c** The level of serum S100A9 in different grades of SMART-COP score in CAP patients. **d** The level of serum S100A9 in different grades of CURXO score in patients with CAP. **e** The level of serum S100A9 in different grades of SMART-COP score in CAP patients. **f** The level of serum S100A9 in different grades of PSI score in CAP patients. All data were expressed as mean ± SEM. *P<0.05, **P<0.01

Serum S100A9 was measured between CAP patients and control subjects. As shown in Fig. 2a, serum S100A9 was increased in CAP patients compared with control subjects. Additionally, serum S100A9 was analyzed among different grades of CAP patients. As shown in Fig. 2b, serum S100A9 was higher in ≥3 score grade than in other grades based on CRB-65 score. According to SMART-COP score, serum S100A9 was higher in 7 ~ 8 score than in other grades (Fig. 2c). Besides, we found that serum S100A9 was higher in severe CAP patients than those in mild CAP patients (CURXO score) (Fig. 2d). What’s more, serum S100A9 was lowest in 0 ~ 1 score and was highest in 3 ~ 5 score on the basis of CURB-65 score (Fig. 2e). Furthermore, the level of serum S100A9 was increased in the grade of III than those in the grades of II and I based on PSI score. Serum S100A9 was highest in the grade of IV (Fig. 2f).

### Correlations of S100A9 with disease severity, blood routine parameters and inflammatory cytokines among CAP patients

The correlations between S100A9 and CAP severity scores were analyzed among CAP patients. We found that serum S100A9 was positively correlated with CURB-65 (*r* = 0.501, *P* = 0.001), CRB-65 (*r* = 0.488, *P* = 0.001), PSI (*r* = 0.567, *P*<0.001), CURXO (*r* = 0.502, *P* = 0.003) and SMART-COP (*r* = 0.475, *P*<0.001) (Table 2). Additionally, the correlations between S100A9 and blood routine parameters were explored. As shown in Table 2, serum S100A9 was positively correlated with WBC (*r* = 0.297, *P* = 0.003), NLR (*r* = 0.274, *P* = 0.006) and MON (*r* = 0.277, *P* = 0.012). Meanwhile, we also observed the correlations between serum S100A9 and inflammatory cytokines. We found that there was positive correlations between serum S100A9 with TNF-α (*r* = 0.248, *P* = 0.001), IL-1β (*r* = 0.273, *P*<0.001) and CRP (*r* = 0.345, *P* = 0.002). Finally, associations between serum S100A9 and CAP severity scores were further analyzed using univariate and multivariate logistic regression. The univariate logistic regression indicated that serum S100A9 was positively correlated with CURB-65 (OR = 1.358; 95% CI: 1.121, 1.652), CRB-65 (OR = 1.223; 95% CI: 1.025, 1.562), PSI (OR = 1.325; 95% CI: 1.056, 1.762), SMART-COP (OR = 1.262; 95% CI: 1.050, 1.462) and CURXO (OR = 1.451; 95% CI: 1.215, 1.864) (Table 3). Confounding factors were controlled and adjusted and the multivariate logistic regression was further performed. These results indicated that serum S100A9 was positively associated with PSI (OR = 1.225; 95% CI: 1.035, 1.562), SMART-COP (OR = 1.212; 95% CI: 1.065, 1.615) and CURXO (OR = 1.116; 95% CI: 1.011, 1.365) (Table 3).

**Table 2:** Correlations of serum S100A9 with disease severity, blood routine examination and inflammatory cytokines

**Disease severity**						
Variables	CURB-65	CRB-65	PSI	CURXO	SMART-COP	
*r*	0.501	0.488	0.567	0.502	0.475	
*P*	0.001	0.001	<0.001	0.003	<0.001	
**Blood routine examination**						
Variables	WBC	Neutrophil	Lymphocyte	NLR	MON	PLR
*r*	0.297	0.135	−0.051	0.274	0.277	0.193
*P*	0.003	0.098	0.621	0.006	0.012	0.081
**Inflammatory cytokines**						
Variables	TNF-α	IL-1β	CRP	IL-6		
*r*	0.248	0.273	0.345	0.057		
*P*	0.001	<0.001	0.002	0.688

**Table 3:** Associations between serum S100A9 and CAP severity scores among CAP patients

Variables	Univariate, OR(95% CI)	*P*	Multivariate, OR(95% CI)^a^	*P*
CURB-65	1.358 (1.121, 1.652)	0.003	0.981 (0.955, 1.007)	0.149
CRB-65	1.223 (1.025, 1.562)	0.005	0.986 (0.970, 1.003)	0.112
PSI	1.325 (1.056, 1.762)	0.001	1.225 (1.035, 1.562)	0.041
SMART-COP	1.262 (1.050, 1.462)	0.001	1.212 (1.065, 1.615)	0.030
CURXO	1.451 (1.215, 1.864)	0.004	1.116 (1.011, 1.365)	0.033

^a^Adjusted for age and sex

### The association between serum S100A9 and the prognosis in CAP patients

**Fig. 3:**
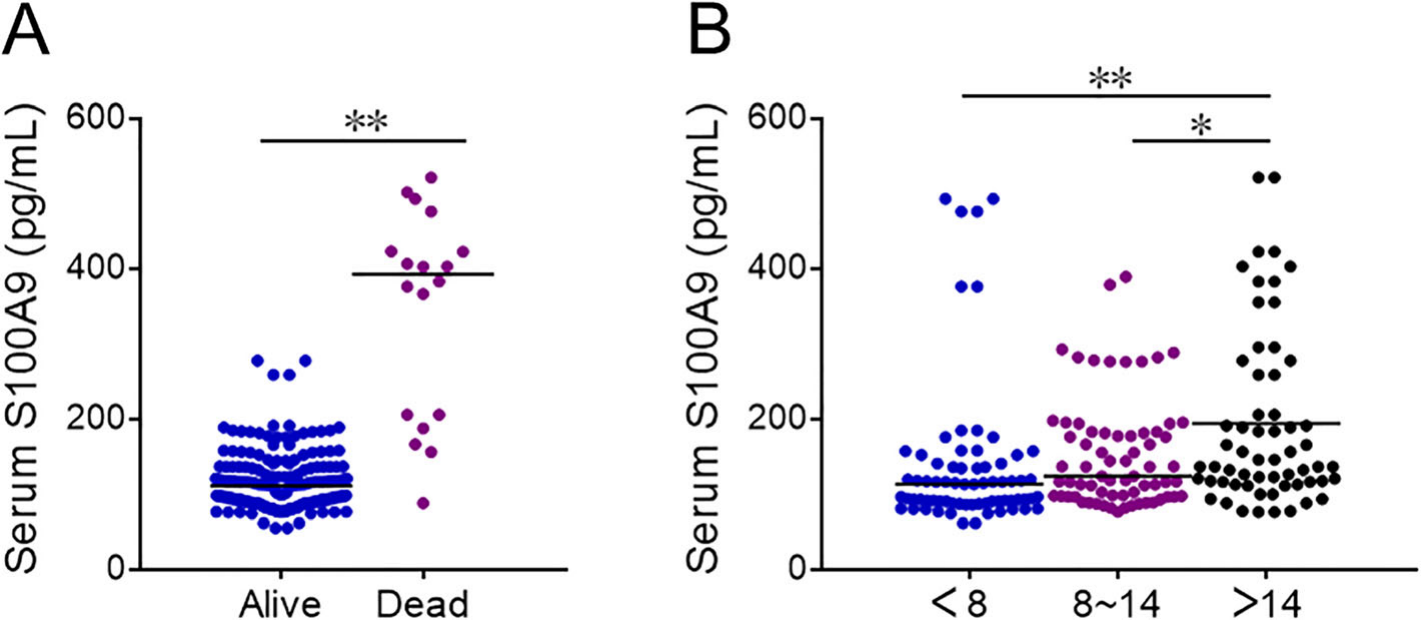
The levels of serum S100A9 in alive and dead CAP patients. **a** and **b** Serum S100A9 was detected with ELISA. **a** The level of serum S100A9 in alive and dead CAP patients. **b** The level of serum S100A9 in different hospital stay of alive patients. All data were expressed as mean ± SEM. *P<0.05, **P<0.01

The level of serum S100A9 were compared between alive patients and dead cases. Eighteen CAP patients were died during hospitalization. As shown in Fig. 3a, serum S100A9 was increased in dead patients than these in alive CAP patients. Moreover, the levels of serum S100A9 was higher in>14 days than these in <8 days and 8 ~ 14 days (Fig. 3b). Besides, the associations between serum S100A9 and the prognosis of CAP patients were analyzed using logistic regression. The univariate logistic regression revealed that serum S100A9 was positively associated with hospital stay (OR = 1.159; 95% CI: 1.062, 1.321) and the risk of death (OR = 1.112; 95% CI: 1.010, 1.336) (Table 4). In order to control confounding factors, the multivariate logistic regression was continued to performing. The results found that serum S100A9 was positively associated with the risk of death (OR = 1.137; 95% CI: 1.023, 1.312).

**Table 4:** Association between serum S100A9 and prognosis among CAP patients

Variables	Univariate, OR (95% CI)	*P*	Multivariate, OR (95% CI)^a^	*P*
Death	1.112 (1.010, 1.336)	0.001	1.137 (1.023, 1.312)	0.012
Hospital stay	1.159 (1.062, 1.321)	0.001	1.001(0.986, 1.016)	0.885

^a^Adjusted for age and sex

### The predictive capacity of serum S100A9 for CAP

**Fig. 4:**
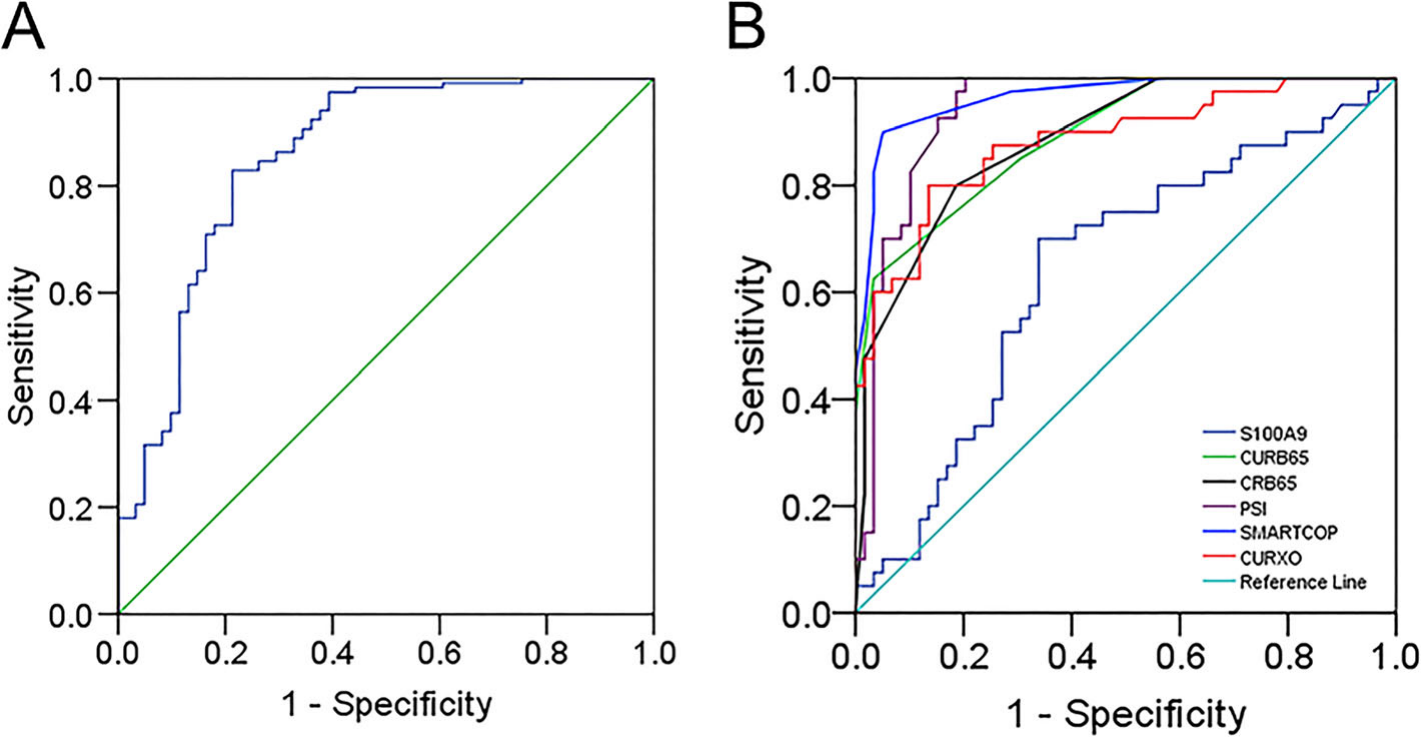
Receiver operating characteristic curves of different predictive biomarkers for CAP. **a** ROC curve was used to assess the diagnostic capacity of serum S100A9 for CAP. **b** ROC curve was used to assess the diagnostic value of different biomarkers (S100A9, CRB-65, CURB-65, CURXO, SMART-COP and PSI) for the severity of CAP

The predictive capacity of serum S1009A was analyzed using receiver operating characteristic area under the curve (AUC). As shown in Fig. 4a, the AUC of S100A9 for CAP was 0.788 (95% CI: 0.699, 0.878). The sensitivity and specificity of S100A9 in the prediction of CAP were 73.5 and 82.5%. Furthermore, the predictive capacity of serum S1009A for severity was analyzed among CAP patients. We found that the AUCs for different biomarkers were as follows: CURB-65, 0.893 (95% CI: 0.832, 0.954); CRB-65, 0.886 (95% CI: 0.823, 0.950); PSI, 0.919 (95% CI: 0.891, 0.987); SMART-COP, 0.916 (95% CI: 0.932, 0.998); CURXO, 0.880 (0.810, 0.951); S100A9, 0.832 (95% CI: 0.715, 0.943) (Fig. 4b). The optimal cut-off value of serum S100A9 level was 213.6 pg/mL with 76.0% sensitivity and 82.0% specificity.

## Discussion

This study mainly analyzed the associations between serum S100A9 with the severity and the prognosis in CAP patients based on a prospective cohort study. The present study mainly found that: (1) Serum S100A9 was elevated in CAP patients; (2) Serum S100A9 on admission was positively associated with CAP severity scores; (3) Serum S100A9 on admission was positively associated with inflammatory cytokines; (4) Serum higher S100A9 on admission elevates mortality and hospital stay in CAP patients.

Earlier studies found that S100A9 is elevated in several diseases, such as neutrophilic inflammation in asthma, insulin deficiency, atopic dermatitis and Parkinson’s disease [17–20]. Moreover, a report from our laboratory indicated S100A8/S100A9 is increased in COPD patients and positively associated with inflammatory cytokines [21]. However, the role of S100A9 in CAP and the associations between serum S100A9 and the severity of CAP were unknown. In the present study, we found that serum S100A9 was elevated in CAP patients. Moreover, serum S100A9 was gradually increased in parallel with the severity of CAP. Further logistic regression analysis found that serum S100A9 was positively associated with the severity of CAP. These results reveal that S100A9 may take part in the pathophysiology process of CAP.

Previous research found that inflammation is increased in CAP patients [30]. In this study, we also found that several inflammatory cytokines were elevated in CAP patients. Not only that, serum S100A9 was positively associated with inflammatory cytokines in CAP patients. Besides, some earlier studies revealed that blood routine indices were changed in CAP patients compared with control subjects [31, 32]. This research found that WBC and neutrophil were increased, lymphocyte was reduced in CAP patients. The ratios of PLR, MON and NLR were increased in patients with CAP. Further correlation analysis demonstrated that serum S100A9 was positively correlated with WBC, NLR and MON. These results suggested that the level of serum S100A9 may reflect the pathophysiologic conditions for CAP in a certain extent.

The association between serum S100A9 and the severity of CAP has been explored. However, the influence of S100A9 on the prognosis of CAP patients remains unclear. The present study analyzed the effect of S100A9 on the death of risk among CAP patients. These results found that the level of serum S100A9 was increased in dead patients on admission. Univariate and multivariate logistic regression analysis verified that serum higher S100A9 on admission elevated the risk of death of CAP patients. Moreover, we found that the higher serum S100A9 was, the longer hospital stay was. Univariate logistic regression analysis suggested that serum S100A9 on admission was positively related with hospital stay in CAP patients. These results proved that serum higher S100A9 on the early stage always indicated a serious prognosis for CAP patients. In order to confirm the predictive capacity, the ROC curve was analyzed. The AUC value of serum S100A9 for CAP was 0.788 (95% CI: 0.699, 0.878). In addition, the predictive quality of serum S100A9 and CAP severity scores for severity were similar. Besides, the predictive power of serum S100A9 was superior to several known biomarkers though literature review [31, 33]. Hence, these results provide additional evidence that serum S100A9 can be used as a better diagnostic biomarker for CAP.

This study mainly explored the relationships between serum S100A9 on the early stage with the severity and prognosis in CAP patients. However, there are several potential flaws in this study. Firstly, all CAP patients and control subjects were recruited from one hospital rather than from multicenter in China. The sample size was relatively small. So, the larger sample size and multicenter studies are needed in the future clinical research. Secondly, this was only a clinical epidemiology research, the mechanism of S100A9 elevation in CAP patients is needed to further clarify with in vitro experiments. Thirdly, S100A9 was only detected in serum, the local levels of S100A9 in sputum, lungs and bronchoalveolar lavage fluid are unknown in CAP patients. Fourthly, S100A9 was only detected in CAP patients. The levels and changes of S100A9 in patients with other pneumonias are unclear. The difference of S100A9 will be compared between CAP patients and other pneumonias’ patients in the next work.

## Conclusion

In summary, this study mainly analyzed the associations between serum S100A9 on admission with the severity and prognosis among CAP patients based on a prospective cohort study. Our results reveal that serum S100A9 is elevated in CAP patients on admission. In addition, serum S100A9 is positively associated with the severity of CAP on admission. We provide evidence that serum higher S100A9 at the early stage elevates the risk of death and hospital stay of CAP patients. Therefore, S100A9 may be regarded as a diagnostic biomarker and useful for the clinical management of CAP in the future clinical practice.

## Data Availability

The datasets used and analyzed during the current study are available from the corresponding author on reasonable request.

## Abbreviations

CAP: Community-acquired pneumonia
WBC: White blood cell
NLR: Neutrophil-lymphocyte ratio
MON: Monocyte-lymphocyte ratio
PLR: Platelet-lymphocyte ratio
TNF-α: Tumor necrosis factor α
IL-1β: Interleukin-1β
CRP: C-reactive protein
